# Multicompartmental Pharmacokinetic Model of Tenofovir Delivery by a Vaginal Gel

**DOI:** 10.1371/journal.pone.0074404

**Published:** 2013-09-11

**Authors:** Yajing Gao, David F. Katz

**Affiliations:** 1 Department of Biomedical Engineering, Duke University, Durham, North Carolina, United States of America; 2 Department of Obstetrics and Gynecology, Duke University Medical Center, Durham, North Carolina, United States of America; University of New South Wales, Australia

## Abstract

**Background:**

Trials of a vaginal Tenofovir gel for pre-exposure prophylaxis (PrEP) for HIV have given conflicting results. Knowledge of concentrations of Tenofovir and its active form Tenofovir diphosphate, at putative sites of anti-HIV functioning, is central to understanding trial outcomes and design of products and dosage regimens. Topical Tenofovir delivery to the vaginal environment is complex, multivariate and non-linear; determinants relate to drug, vehicle, dosage regimen, and environment. Experimental PK methods cannot yield mechanistic understanding of this process, and have uncontrolled variability in drug sampling. Mechanistic modeling of the process could help delineate its determinants, and be a tool in design and interpretation of products and trials.

**Methods and Findings:**

We created a four-compartment mass transport model for Tenofovir delivery by a gel: gel, epithelium, stroma, blood. Transport was diffusion-driven in vaginal compartments; blood concentration was time-varying but homogeneous. Parameters for the model derived from *in vitro* and *in vivo* PK data, to which model predictions gave good agreement. Steep concentration gradients occurred in stroma ≤8 hours after gel release. Increasing epithelial thickness delayed initial TFV delivery to stroma and its decline: *t_max_* increased but *AUC* at 24 hours was not significantly altered. At 24 and 48 hours, stromal concentrations were 6.3% and 0.2% of *C_max_*. Concentrations in simulated biopsies overestimated stromal concentrations, as much as ∼5X, depending upon time of sampling, biopsy thickness and epithelial thickness.

**Conclusions:**

There was reasonably good agreement of model predictions with clinical PK data. Conversion of TFV to TFV-DP was not included, but PK data suggest a linear relationship between them. Thus contrasts predicted by this model can inform design of gels and dosage regimens in clinical trials, and interpretation of PK data. This mass transport based approach can be extended to TFV conversion to TFV-DP, and to other drugs and dosage forms.

## Introduction

Vaginal application of topical microbicidal molecules, that inhibit sexual transmission of HIV, is an important modality that is being developed to combat the AIDS pandemic [1]–[3]. Several types of delivery systems are being evaluated for these molecules, including gels, films, dissolving tablets, fiber meshes and intravaginal rings [4]–[8]. The successes of any of these products depend upon the intersections of their capabilities for delivery of specific drugs with user adherences to designated product use. After failures of early microbicide gel products, a successful Phase 3 trial was conducted of a hydroxyethylcellulose (HEC) gel loaded with a 1% concentration of the anti-retroviral molecule Tenofovir (TFV), and applied vaginally at a 4 mL volume (the CAPRISA 004 Trial) [9], [10]. However, a follow up trial with the same gel and applied volume failed to demonstrate such efficacy (the VOICE Trial) [11]. Recently reported analysis of product usage in VOICE indicated that there was very poor adherence to the designated gel application schedule [12]: this was to be daily, regardless of sexual activity. In contrast, CAPRISA 004 used a before-and-after gel dosage regimen. At present, the failure of anti-HIV efficacy in VOICE is attributed in large part to poor adherence to the daily dosing schedule, although further analyses are underway. In any event, it is possible that the different dosage regimens in the two trials (when gel was actually used) led to differences in the time and space concentration distributions of Tenofovir at the mucosal sites where it is believed to act. These differences could have extended, also, to effects of missed gel applications on mucosal drug concentrations. More fundamentally, our knowledge of the determinants of pharmacokinetics (PK) for human vaginal delivery of Tenofovir, and candidate anti-HIV microbicidal molecules in general, is limited. As a consequence, conclusions regarding mucosal drug concentration distributions between the CAPRISA 004 vs. VOICE trials cannot definitively be drawn.

The goal of the present study was to contribute to the limited knowledge of the determinants of microbicide PK by introducing a mechanistic compartmental analysis framework within which to predict the time and space histories of the concentrations of drugs delivered to the vaginal environment by gels. These concentration distributions are central to the anti-HIV efficacies of the applied drugs. They depend upon details of the drug, gel, dosage regimen and the vaginal environment. We have begun with a gel vehicle because there are substantially more data about this dosage form than any other. However, our approach is extendable to the other dosage forms being evaluated. Although the details may be different, the fundamental principles of mass transport remain the same. Thus, this type of computational framework can be a tool with which to ask biologically and pharmacologically meaningful questions about how vaginal drug delivery may vary as those details vary, and thus be optimized to achieve target drug concentrations at sites of anti-HIV activity.

Our approach embodies the principles of mass transport theory. The focus here is on drug concentration distributions across the vaginal mucosa, within which leading microbicide drugs act against HIV. In particular, we develop and apply a model for gel delivery of Tenofovir. This is the contemporary microbicide drug about which the most is known, biologically and clinically [13]–[16]. Tenofovir acts against semen-derived HIV after interacting with CD4-positive host cells (e.g. T helper cells, macrophages, dendritic cells) which the virions may access [17]. In those cells, which exist in the mucosa, it is converted to Tenofovir diphosphate (TFV-DP). This is bioactive in inhibiting the HIV DNA transcriptase process, which is essential to HIV replication and the onset of infection [16], [17]. Other current leading microbicide drugs, e.g. dapivirine MIV150 and IQP-0528, are similar to Tenofovir in that that they are also reverse transcriptase inhibitors. However, these other drugs are direct RTIs, in that they do not require a transformation reaction within host cells in order to exhibit potentially prophylactic action [18]–[22]. Tenofovir is the drug about which the most is known – in terms of PK data (human, animal) and actual Phase 3 efficacy testing. It is relatively water soluble. It does enter cells that it encounters in mucosa, within which it is phosphorylated to TFV-DP; within the vaginal mucosa these include the cells of the upper stratified squamous epithelium (which is several cell layers thick) and the host cells in the lower stromal layer. There is ongoing transport of TFV into these cells, followed by phosphorylation to TFV-DP. It is believed that cells tend to retain the TFV-DP, but TFV is free to leave these cells. Overall, there is a linear correlation between TFV and TFV-DP concentrations, with TFV-DP about 5%–7% of the TFV concentration [15]. The overall distribution and transport of TFV through the mucosa are thus a combination of paracellular and transcellular mechanisms. In PK studies, measurements of TFV and TFV-DP in tissue biopsies do not currently distinguish between intracellular and transcellular drug. As such, they do not directly measure its bioavailability, and they produce a concentration that is a volume average over the epithelial thickness and a portion of the stromal layer (see below). Nonetheless, biopsies are deemed valuable in confirming and quantifying delivery of drug to target compartments. In our initial model here, we do not distinguish between transcellular and intracellular drug. We define the epithelial and stromal compartments as homogeneous, and assume that there are effective diffusion coefficients for drug transport through each (obtained from *in vitro* permeability experiments, see below). Thus, the model predicts the types of measurements performed in PK studies; its predictions can be compared to them and be used to ask questions about the multiple factors that govern Tenofovir delivery to the vaginal mucosa.

**Figure 1:**
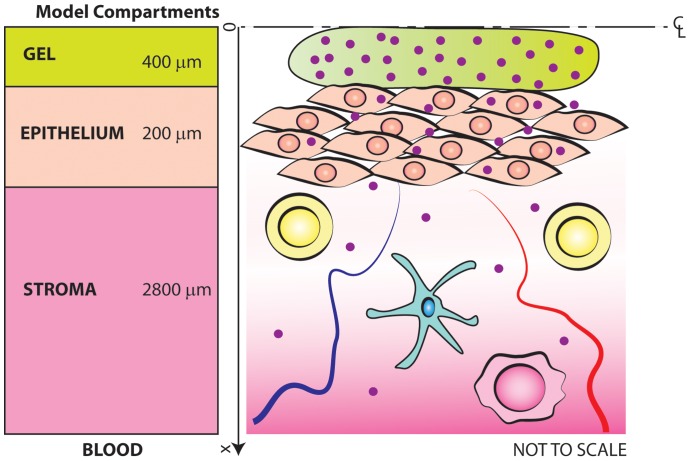
Drawing of structure of vaginal mucosa. This shows the gel layer over the epithelial surface. The small red circles are drug molecules. This view is the lower half of the complete side view of the lumen of the vaginal canal. A bilaterally symmetrical set of layers exists above those shown here.

Figure 1 is a schematic drawing of the lumen and mucosa of the human vagina. The thickness of the stratified squamous epithelial layer varies with respect to the phase of the menstrual cycle, and is largest (as much as ∼400 µm) during the 5 day peri-ovulatory period [23]. The stroma (or lamina propria) is made up of connective tissue, host cells, vasculature and lymphatics. The human vaginal stroma is about 2–3 mm thick, and the network of blood vessels (which free drug may enter and then be cleared) occupies about 10% of the total stromal volume [24]. The concentration of stromal CD4-positive cells has not been objectively quantified, but is believed to be low (e.g. ≤10^6^ cells/mL). Thus, these target cells are widely spaced; e.g. for a concentration of 10^6^/mL, the average spacing between them (if distributed randomly) is of the order of 100 µm or greater [25]. This simplifies analysis and interpretation of Tenofovir transport through the stroma, since a very small proportion of the entering molecules are converted to Tenofovir diphosphate in host cells. Therefore, this conversion has a small effect on Tenofovir concentration distribution overall. Because there is a linear correlation between concentrations of Tenofovir and Tenofovir diphosphate, inferences about contrasts in TFV-DP can be drawn from measurements of TFV [15]. There is also ambient vaginal fluid present (not shown in Figure 1); total volume of this fluid, which probably tends to collect in the interior vaginal fornix [26], is believed to be <1 mL at any time and is maximum at midcycle [27], [28].

Contemporary pharmacokinetic studies assess drug concentrations in vaginal tissues by computing values in homogenized small biopsies [15], [29]. These biopsies vary in thickness, but always include the epithelial layer and about 2–3 mm of the stromal layer. Thus, they cannot delineate partitioning of drugs between the epithelium (which has no vasculature that clears drugs) and the much thicker stroma (which contains the majority of HIV host cells and which does clear drugs to the blood stream), and the potentially sharp concentration gradients across epithelium and/or stroma (see below). As a result, the relationship between drug concentration in a biopsy and its concentration at its target prophylactic site – for Tenofovir this is believed to be primarily the stroma – is not unique, and will depend upon uncontrolled factors such as thicknesses of the epithelium and the biopsy, and the time at which the biopsy was taken in relation to the history of gel application.

Clearly, the rational design of any microbicide gel product would benefit from objective understanding of how gel properties, applied volume and dosage regimen govern the time and space histories of drug concentrations in target tissues and fluids, e.g. the vaginal stroma. Computational models of drug delivery are widely used in pharmacology [30], and could contribute to such understanding. In the context of the HIV therapy-microbicides field, our approach is similar to the traditional one that has been applied [5] in that both implement conservation of drug mass principles for an ensemble of contiguous compartments (tissue, blood, etc.). However, in our approach drug transport within compartments is characterized, since this mechanistically governs rates of exchange between compartments as the drug migrates out from its vehicle, into and through body fluids and tissues, and eventually into the blood stream. In contrast, the traditional approach tends to treat each compartment as containing a spatially homogeneous (but time varying) concentration, with rate constants governing drug loss within each compartment as well as exchange between compartments. Earlier, we applied conservational of mass principles to create a computational model of Cyanovirin-N (CV-N) delivery by a vaginal gel into semen [31]. CV-N can act against HIV within the fluids of the vaginal lumen by colliding with, binding and then neutralizing HIV virions. In this two-compartment model, CV-N molecules (initially in a gel layer) and HIV virions (initially in a semen layer above the gel layer) moved by diffusion and collided; local HIV neutralization kinetics was deduced. The two layers were taken to be of uniform (but different) thicknesses, and to cover the entire vaginal epithelium. Because kinetic parameters for both HIV binding and neutralization were available for CV-N, this model could be implemented to predict the time and space dependent concentration distributions of both infectious and neutralized virions. Application of the model focused upon dependence of HIV neutralization on properties of the gel layer, viz. the diffusion coefficient of virions in the gel, and the thickness of the layer. Undiluted gels substantially retard viral diffusion, but gel dilution by ambient vaginal fluids can lead to increased diffusion [32]. The model was helpful in developing an initial mechanistic understanding of the significance of this effect, delineating tradeoffs between viral diffusion coefficient within, and thickness of the gel layer in achieving target neutralization of the invading HIV virions. However, this model did not address drug delivery into the vaginal mucosal tissue, which is relevant to the more recent candidate microbicide drugs. We also used mass conservation principles to create a computational model of Dapivirine delivery from an intravaginal ring to the surface of the vaginal epithelium [27]. This model embodied diffusion and also convection (by vaginal fluid) drug transport processes. Its application included the prediction that convection in vaginal fluid plays a paramount role in drug delivery by a ring. However, this model did not include drug transport into the vaginal mucosa.

The present work begins the process of creating and applying mechanistic models for microbicide drug delivery from vaginal microbicide dosage forms, down into the vaginal mucosa, with clearance to the blood stream in the stroma. We have sought to create as simple as possible a model for a gel, that predicts concentration distributions which are quantitatively comparable to concentration measurements in the limited amount of human PK data for the 1% Tenofovir gel that was applied in past and recent clinical trials. There are two studies in which vaginal fluid, biopsy tissue and blood were collected after different dosing protocols of 4 mL of the 1% Tenofovir gel. Tenofovir and Tenofovir diphosphate concentrations were measured in all three matrices. In one study (termed the CONRAD study, [15]), data were obtained at 0.5, 1, 2, 4, 6, 8 or 24 hours post a single gel dose, and also for twice daily dosing (at 12 hour intervals for 2 weeks). In the other study (named MTN-001, [29]) one of the protocols was gel dosing once per day, designated at bedtime, for 6 weeks. Sampling was performed at ostensibly steady state conditions at 3 and 6 weeks. In the US arm of the study, the specimens were taken either pre-dose or at 2, 4 or 6 hours after the most recent vaginal gel dose. These two human PK studies provide data that we reference in determining several parameters in our model, and also in initial validation of its predictions. We consider spatial TFV concentration distributions across the epithelium and stroma over 48 hours after a single gel dose, to delineate the build-up of drug from the time of application, and the much lower levels that would occur if, say, a daily (at 24 hours) dose were missed. We also vary the thickness of the epithelial layer and rate of gel dilution due to varying amounts of vaginal fluid, commensurate with normal variations associated with the phase of the menstrual cycle and the onset of menopause; and we vary the thickness of the biopsy specimen (measurement within which is predicted by our model) to bracket what occurs in real tissue sample collection and its effect on the relationship between concentrations measured in a biopsy and actual concentrations in the stromal compartment.

In addition to the two human PK studies with the Tenofovir gel, there have been two related animal PK studies, one in macaques [33] and one in rabbits [34]. The earlier macaque study applied 0.6 mL of gel per kg of body weight (i.e. about 3–4 mL) of HEC-based Tenofovir gels formulated at three concentrations (0.5%, 1% and 5%). It was stated that, apart from the varying Tenofovir concentrations, these gels had the same composition as the human clinical 1% gel. The rabbit study compared 1 mL applications of two 1% Tenofovir clinical gels, the original clinical one (that contains 20% glycerol and is hyperosmotic) and a reduced glycerol one (5%, giving rise to lower osmolality). These two studies addressed important contrasts in topical Tenofovir delivery by vaginal gels. However, they were not allometrically scaled to humans, and thus their results cannot directly be related to quantitative PK data in humans.

## Materials and Methods

### Geometry of Model

The vaginal canal has a relatively flat cross section, with a very thin undistended height vs. width and length. Its dimensions are believed vary with respect to many factors, e.g. parity and BMI. Here we use a net surface area of 100 cm^2^, which is a key morphometric measure governing net drug delivery from gel to the epithelial surface in our model [35], [36]. The microbicides field has experimented with gel volumes ranging from 2.5–5 mL, and current thinking is that an upper bound is 4 mL; higher volumes would be prone to leakage that could diminish user adherence to designed gel use. A volume of 4 mL was used in the CAPRISA 004 and VOICE trials. If distributed uniformly along a vaginal canal of surface area 100 cm^2^, this gel would have a thickness of 800 µm (between upper and lower vaginal walls); that thickness is small compared to the linear dimensions of the canal. Thus, we use a rectilinear geometry, with top-to-bottom symmetry about its centerline. We assume complete coating of the epithelial surfaces with a uniform thickness, and one-dimensional diffusional transport of the drug from the gel layer into the epithelial and stromal layers of the mucosa cf. Figure 1. These are the geometric assumptions we used in our initial modeling of microbicide drug release by a vaginal gel coating layer [31]. The analysis here does not address the consequences of semen deposition and mixing with gel. Rather, we present a computational framework that characterizes the mucosal drug distribution that would exist prior to the time of semen deposition.

### Governing Transport Equations for Gel, Epithelium and Stroma

We pose a system of coupled unsteady diffusion equations that express conservation of mass for the drug in the gel, epithelial and stromal layers (Equations 1 a,b,c). These are coupled to an equation for the blood compartment, Equation 3 (see below).
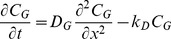
(1a)

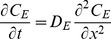
(1b)

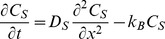
(1c)


**Equations 1. Governing equations for API transport from a gel layer down into epithelial and stromal layers.** The symbols *C* and *D* denote the local drug concentration and diffusion coefficient, respectively. The subscripts *G*, *E* and *S* denote values in the gel, epithelium and stroma, respectively. The symbols *k_D_* and *k_B_* are first order loss rate constants, characterizing effects of dilution of the gel layer and loss of drug to the bloodstream and lymphatics in the stroma, respectively. The origin of the spatial dimension *x* is the centerline of the gel, and *x* is positive from the origin through the gel into the tissue. At time *t* equal to zero, the gel contains its loaded concentration of drug *C_o_*.

Equation 1a expresses conservation of mass for the gel layer. We do not include a discrete layer for ambient vaginal fluid. Rather, we assume that it is mixed rapidly into the gel, and account for this as a time-dependent spatially-homogeneous process with a specified a rate coefficient *k_D_* [37]. We also assume that the change in gel volume due to dilution is small, which is justified because the amount of vaginal fluid present is small compared to the gel volume. The small change in gel thickness due to the slight increase in gel volume from uptake of vaginal fluid has a negligible effect on drug transport into the epithelial and stromal layers (computations not shown); this is due to the very large drop in diffusion coefficient between the gel and the tissue layers, which results in a relatively flat concentration profile in the gel layer (see below). The rate constant *k_D_* also accounts for loss of drug in the gel layer due to leakage.

Equation 1b models conservation of mass of drug within the epithelial layer. This assumes that net transport through the layer can be characterized as a single diffusion process, as discussed in the Introduction above. Equation 1c gives conservation of mass of drug within the stromal layer. We model loss of drug to the vasculature and lymphatics within that layer as a first order process, with net rate constant *k_B_*. This assumes uniform distribution of capillaries and lymphatics throughout the stroma, and that the volume fraction of these capillaries is relatively small (it is about 10%) [24]. Because drug transport in the blood is fast compared to diffusion in the tissue outside it, its mass transfer into the blood is effectively proportional to local tissue concentration (rather than the difference between the tissue and blood concentrations [38]); further, there is negligible transport of drug from capillaries back to tissue. Thus *C_S_(x,t)* in the stromal layer depicts drug concentration in tissue only, excluding the concentration within the stromal vasculature.

### Boundary and Initial Conditions

The boundary and initial conditions for the governing equations are given in Equations 2. We introduce partition coefficients at the boundaries between the gel and epithelium, and between the epithelium and stroma. We assume zero concentration at the outer margin of the stroma, noting that this boundary condition has a very small effect on the behavior of the solution.
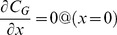
(2a)

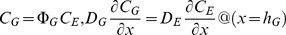
(2b)

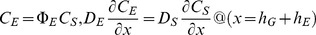
(2c)

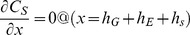
(2d)

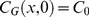
(2e)

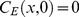
(2f)

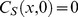
(2g)


**Equations 2. Boundary and initial conditions for the system of transport equations.** The variables *C*, *D*, and *h*, denotes the concentration, diffusion coefficient, and layer thickness respectively for each layer. The layers, indicated by subscripts *G*, *E*, and *S*, are for the gel, epithelium, and stroma. The symbols 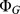 and 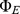 are partition coefficients at the gel-epithelial and epithelial-stromal interfaces, respectively.

### Conservation of Mass for the API in the Blood Compartment

We model the blood as a single compartment with spatially homogeneous drug concentration. Drug enters through the stromal vasculature. We use a single first order kinetic term, with volumetric rate *k*_L_, to account for the net loss rate of drug from the blood compartment. Neglecting other drug interactions in the blood compartment, its concentration can thus be expressed by the following conservation of mass equation.
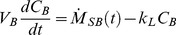
(3a)


**Equation 3a. Conservation of mass equation for drug concentration in blood plasma.** Symbols are defined in the text.

Here *V_B_* is the volume of distribution of the blood compartment, *C_B_* is the concentration in the blood and 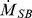 is the volumetric rate of drug entering blood from the stroma – this is the volume integral of the second term on the right hand side of Equation 1c (i.e. drug concentration in the stroma times the kinetic rate of transfer between the stroma and the blood). Equation 3 is solved by integration by parts, yielding.
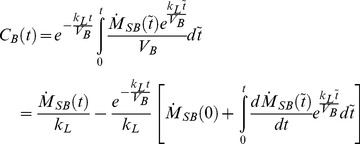
(3b)


**Equation 3b. Solution to differential equation for concentration in the blood compartment.** Here we have assumed zero initial concentration of drug in the blood at the time of gel insertion. Note that the expression for the time-dependent drug concentration in the blood contains three terms: a pure input term 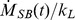 (from the blood vessels in the stroma into which drug passes) minus a simple exponential decay (with loss coefficient *k_L_*) and a second term which is an interaction between the input and first order loss effects.

### Choosing Parameters in the Model

There are a number of parameters in this model (Table 1). As standard conditions, we take the epithelial and stromal layer thicknesses as 200 µm and 2.8 mm respectively [23]. Thickness of the gel layer in the computations here is 400 µm. We note, again, that this parameter is half the total thickness of coating across the full thickness of the lumen because drug transport is bilaterally symmetrical about the centerline of the gel coating (see Introduction). For a typical human vaginal canal of surface area 100 cm^2^, the 400 µm value thus corresponds to complete coating of the canal by a gel volume of 4 mL. Values of some of the remaining parameters can be estimated based on direct experimental data for Tenofovir. These include the diffusion and partition coefficients. Based on its molecular weight (287 [39]), the effective radius of Tenofovir is estimated to be 0.3815 nm (www.molinspiration.com). The resulting diffusion coefficient in water, from Stokes-Einstein theory, is 8.5×10^−6^ cm^2^/s [38]. Diffusion coefficients of molecules in vaginal microbicide gels have been sparsely measured to date. Coefficients for fluorescein (molecular weight 322 Da) and a 10 kDa dextran were measured in three gels, one of which (termed PCS) is very similar to the clinical Tenofovir gel focused upon here [40]. These measurements also confirmed the accuracy of the computed diffusion coefficient from Stokes Einstein theory. Values of diffusion coefficients in undiluted gel, normalized by their diffusion coefficients in water, were 0.68 for fluorescein and 0.57 for the dextran. The normalized diffusion coefficient for fluorescein increased to 0.8 after 1∶1 gel dilution with water. On the basis of these data, we have used the value 6×10^−6^ cm^2^/sec for the diffusion coefficient of Tenofovir in the gel. Tenofovir permeability has been measured in human cervico-vaginal tissue explants, after release from the 1% clinical gel [13], [41]. For a typical tissue thickness of 0.5 mm, the median value for these data gives an estimate of the product [partition coefficient (at gel-tissue interface)×diffusion coefficient] of about 5×10^−8^ cm^2^/sec. Tenofovir is not a hydrophobic drug [42]. The pH of the extracellular fluid in the mucosa is about 7.4 and Tenofovir concentrations therein are relatively low. Thus, Tenofovir solubility in the mucosa is expected to be relatively high, and we take a partition coefficient of unity. Thence, we assume equal diffusion coefficients within epithelium and stroma as 5×10^−8^ cm^2^/sec. The value for *V_B_*, volume of distribution in the blood compartment was taken as 75 L [17].

**Table 1:** Standard values of parameters used in the model.

Parameter	Symbol	StandardValue
Gel Diffusion Coefficient (cm^2^/s)	*D_G_*	6×10^−6^
Epithelium Diffusion Coefficient (cm^2^/s)	*D_E_*	5×10^−8^
Stroma Diffusion Coefficient (cm^2^/s)	*D_S_*	5×10^−8^
Gel/Epithelium Partition Coefficient	*φ_GE_*	1
Gel Thickness (cm)	*h_G_*	0.04
Epithelium Thickness (cm)	*h_E_*	0.02
Stroma Thickness (cm)	*h_S_*	0.28
Rate Constant due to Dilution in Gel (hr^−1^)	*k_D_*	0.551
Rate Constant to Blood Vessels in Stroma (hr^−1^)	*k_B_*	0.122
Rate Constant for Loss in Blood (hr^−1^)	*k_L_*	1.19
Initial Concentration (ng/ml)	*C_0_*	10^7^
Blood plasma volume (L)	*V_B_*	75

**Figure 4:**
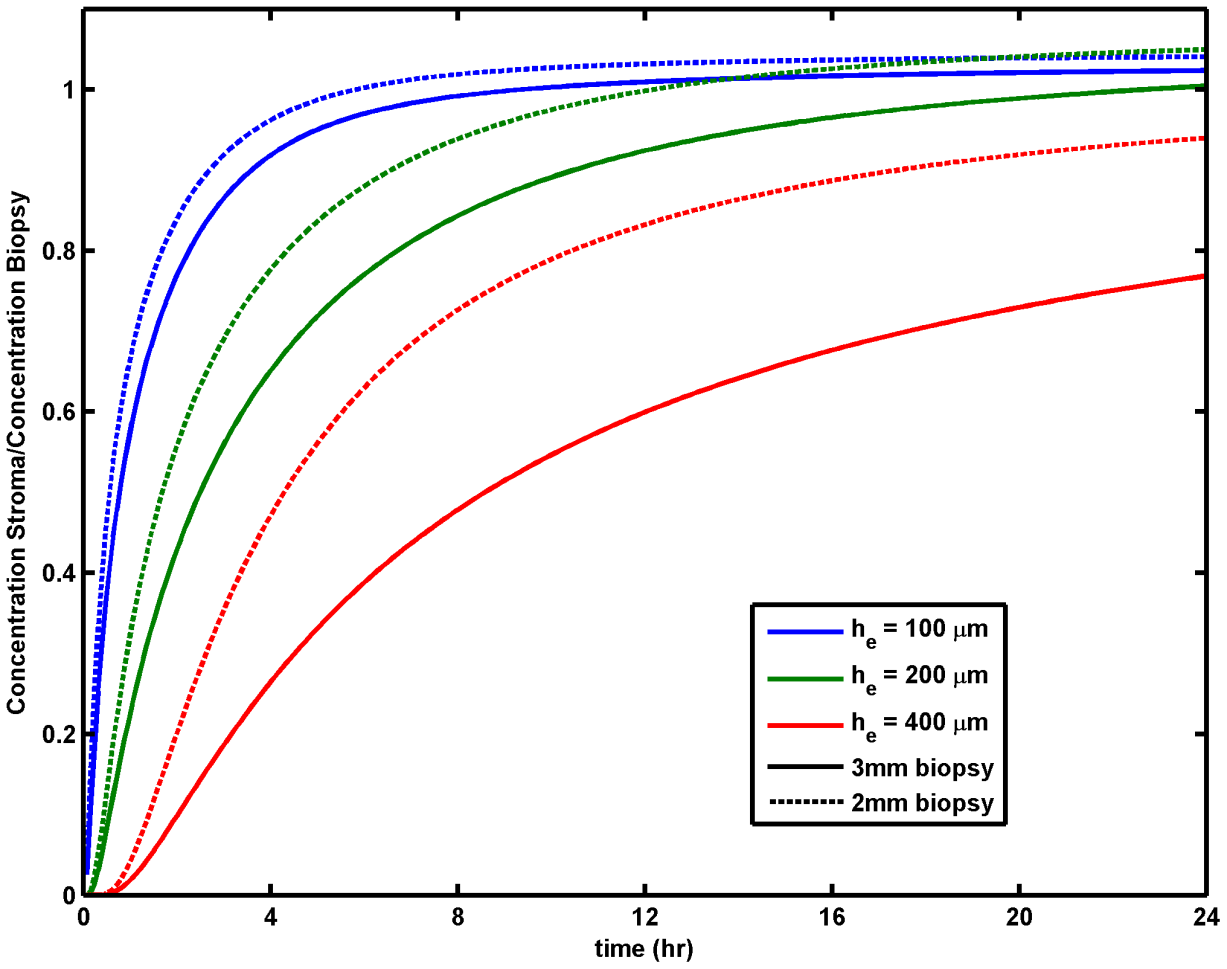
Ratio of drug concentration in the stromal layer to concentration in a simulated biopsy. Standard conditions for gel and stromal thickness. This shows effects of differences in epithelial and biopsy thicknesses.

There are three additional parameters in our compartmental model, the three rate constants for gel dilution/leakage, drug loss to the vasculature and lymphatics in the stroma, and drug loss from the bloodstream: *k_D_*, *k_B_* and *k_L_*, respectively. There are no data for *k_D_* and *k_B_* which correspond to our model. For oral dosing of Tenofovir (given as Tenofovir disoproxil fumurate), there have been a number of PK studies, from which values of a clearance rate constant in the blood compartment were calculated using standard PK models [15], [30]. However, the value of *k_L_* in our model does not strictly correspond to those results because the input to the blood compartment from the stroma here is different (see equation 3b). Consequently, the values of the three rate constants for Tenofovir in our compartmental model were chosen based on fits of our results to the PK data set for human vaginal application of 4 mL of the 1% Tenofovir gel in a single dose [15]. That study computes standard PK summary parameters and gives plots of Tenofovir concentrations in aspirated vaginal fluid, vaginal biopsies and blood plasma vs. time after application of single or two successive gel doses (cf. Figure 4A in that paper). Values of *C_max_*, *C_24h_* and *AUC* are also given for those compartments (cf. Table 3 in that paper). We computed values of the three rate constants that gave a best fit of computationally predicted values vs. the experimentally measured ones. A cost function was defined as the sum of the normalized square differences between predicted and measured values of C_24h_ and the value of concentration at the experimentally measured t_max_ values in the tissue and blood compartments. Best-fit parameters were obtained using the Nelder-Mead Simplex Method (MATLAB algorithm “fminsearch”; MathWorks [43]), following initial guesses based on simple kinetic models for each of the three parameters: for *k_D_* we assumed complete dilution of a specified vaginal fluid volume in 24 h; for *k_B_* we based our guess on the slope of the data in the tissue compartment in the Schwartz et al PK study; and for *k_L_* we based our guess on the slope of the data in the blood compartment in the same study. We did not include reference values for aspirated vaginal fluid in these computations because this fluid was pipetted out from the fornix, and does not directly correspond to the gel compartment in our model. The Tenofovir concentration in a biopsy is a volume average in a piece of tissue that includes the epithelial layer and about 2–3 mm of the stromal layer. Therefore, the simulation of a biopsy in our computations was the spatial average of concentrations in the combined epithelial and stromal compartments. Direct comparisons of our predicted and the experimentally measured results are given below in the Results section.

### Numerical Solution of the Equations

The equations were solved using MATLAB software. They were first converted into difference equations using the forward difference method. The spatial dimension was discretized into approximately 500 points. The boundary conditions were rewritten as difference equations. MATLAB’s built-in “ode15s” function, with default parameters, was used for solving the equations. The stiff solver was implemented to reduce computation time, since the large drop in concentration at the boundary initially presents a large derivative when calculating the flux. We note that the equation solver can be paused (in future enhancements of this model) in order to simulate instantaneous dilution due to semen deposition or repeated application of additional gel. Drug concentration in the blood serum was calculated after the concentrations in the stroma and epithelium were obtained, using the MATLAB “ode” solver. Spatial average concentration values for each compartment were computed using the trapezoidal rule for numerical integration over concentration profiles at each time step.

## Results

### Spatial Drug Concentration Profiles in Compartments at Different Times

**Figure 2:**
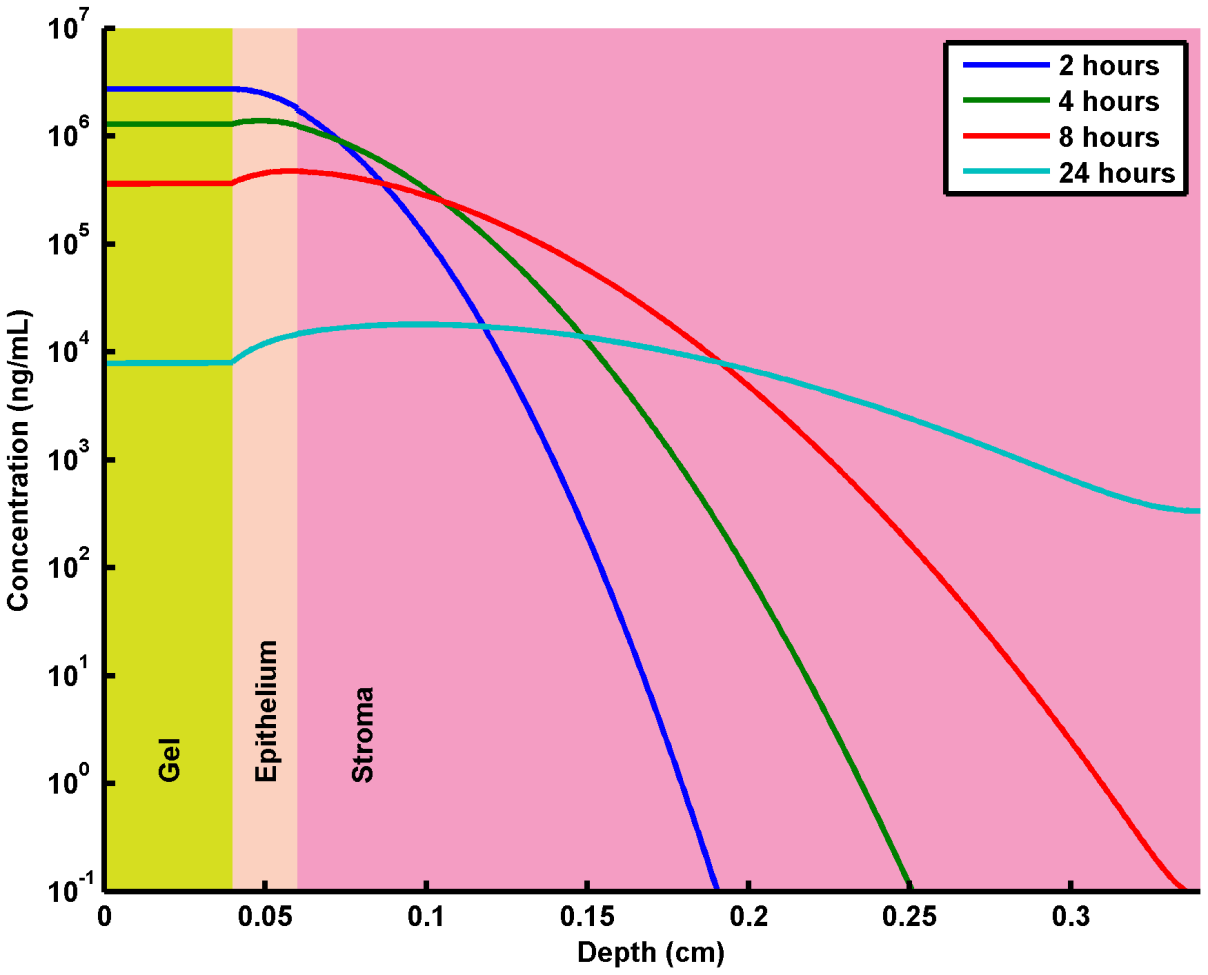
Spatial concentration profiles of Tenofovir. Standard conditions for gel, epithelial and stromal thicknesses. These are at different times in the half space between the centerline of the gel compartment layer and the epithelial and stromal layers.

The fundamental outputs of this model are Tenofovir concentrations as functions of time and position (depth) within the gel, epithelial and stromal compartments, and concentration (volume averaged) as a function of time in the blood compartment. We found that increasing the number of computational grid points above 500 altered results by ≤1%; thus this grid size was deemed asymptotically valid for the computations. Figure 2 shows concentration profiles in the contiguous gel, epithelial and stromal compartments, at 2, 4, 8, 24 and 48 hours after the onset of drug release from the gel. In practice, 24 hours has been an upper bound on the interval between successive gel applications in clinical trials. We have included the 48 hour time point to assess effects of missing a gel application. The concentration of drug across the gel layer is nearly constant, a consequence of the two log drop in the diffusion coefficient from the gel to the epithelial tissue. By 24 hours, the concentration in the gel is about 2.5 logs lower than its initial value. Local concentration initially decreases with distance down into the epithelium. Over time, however, gel leakage and dilution become increasingly significant in reducing concentration across the gel layer, so that concentration actually increases slightly with depth down into the epithelium. There are time-dependent concentration gradients in the stromal compartment. These are initially steep, with about 2 log drops through the depth of the stroma at times up to 4 hours. By 24 h there is about a 1 log drop. At 48 hours, the concentration in the stroma is much flatter, and has dropped a further 2 logs in the upper 2 mm of that compartment.

### Average Concentrations in Compartments vs. Time

**Figure 3:**
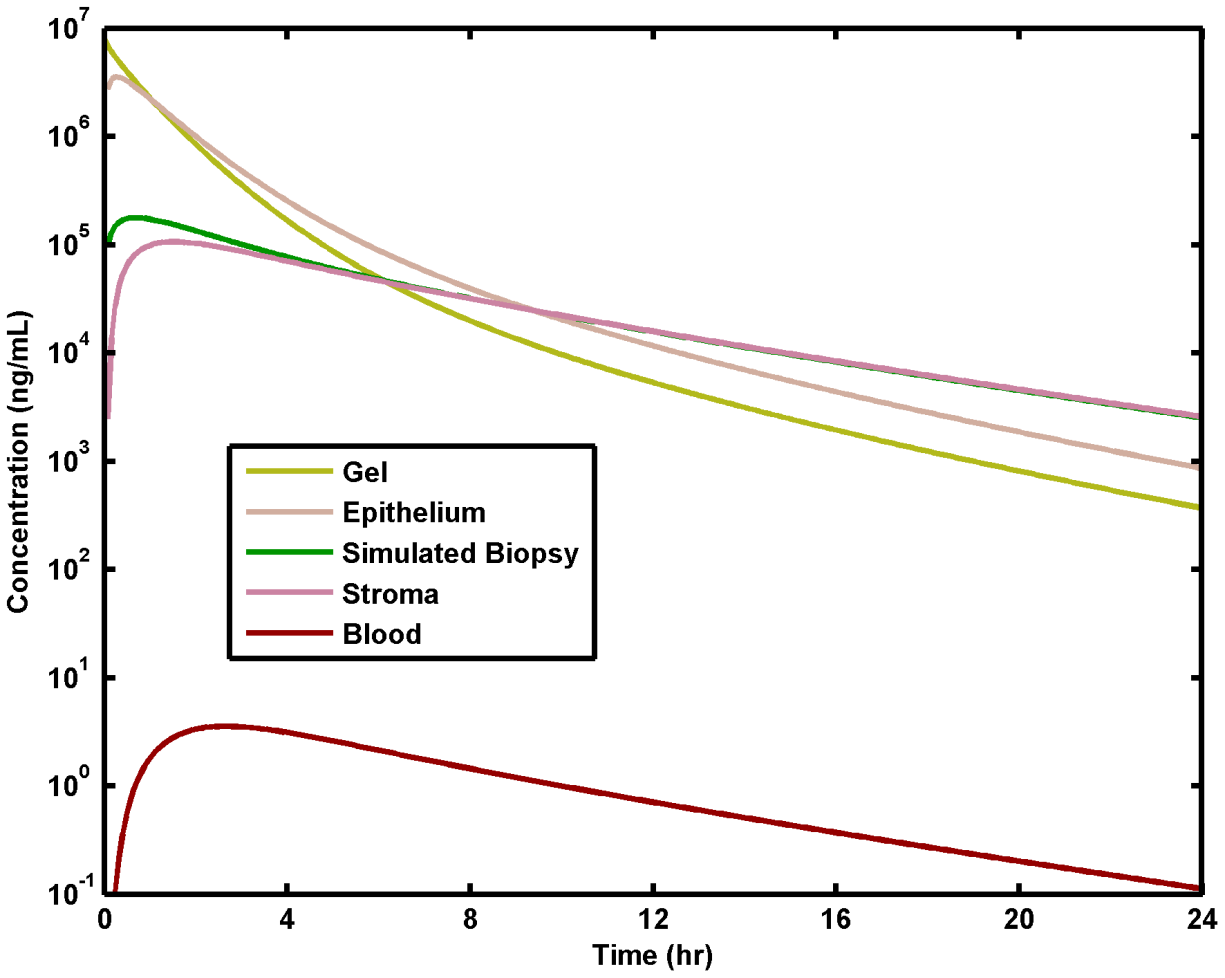
Average concentrations in different compartments vs. time. Standard conditions for gel, epithelial and stromal thicknesses. These are spatial averages through the depths of the compartments. The simulated biopsy is the spatial average of combined epithelial and stromal compartments.

Figure 3 shows depth-averaged concentrations of Tenofovir in each compartment, and also the average over the epithelial and stromal compartments combined (which simulates what would be measured in a biopsy). These depth-averaged values correspond to volume averages, since we are implementing a 1D model for the transport process. Both in the tissue and blood plasma, the maximum concentration is achieved at around 4 hours, after which there is a nearly linear decline in concentration on the log scale, i.e. exponential loss. The concentration in the gel is fairly close to the concentration in the epithelium, due to the small thickness of the epithelial layer and the non-steep spatial gradients in both compartments (Figure 2). There is about a 2 log drop in concentration from the gel to the stroma, about a 1.5 log drop to the simulated biopsy, and then about a 5 log drop to the blood plasma. In the stroma, maximum, 24 hour and 48 hour average concentrations are, respectively: C_max_ = 1.01×10^5^ ng/mL, C_24_ = 6.93×10^3^ ng/mL and C_48_ = 2.70×10^2^ ng/mL.

### Pharmacokinetic Summary Measures

Table 2 gives computations by the model of summary pharmacokinetic parameters for a single application of 4 mL of gel. The *AUC* values are over 24 hours following gel application. Corresponding values for the human PK data from the Schwartz et al study are given (parentheses and italics) next to each of the computational results. Our computations for the gel compartment do not correspond to experimental values for aspirated vaginal fluid, and so they are not included in this comparison table. Our computed values of *C_max_* and *C_24 h_* in biopsies and blood are very close to the experimental data. Our values of *AUC* tend to be higher than the computed values from the PK data, particularly for the biopsy. This could derive from the fact that we computed *AUC* by integrating values of concentration over time as an effectively continuous variable, with a very small time step; in contrast the experimental computation of *AUC* is based upon a small number of values of concentration at unevenly spaced times.

**Table 2:** Summary pharmacokinetic parameters from computations by model for single application of 4.

	*t_max_* (hr)	*C_max_* (ng/mL)	*C_24 h_* (ng/mL)	*AUC* (ng/mL*hr)
**Biopsy**	1.3 *(2)*	2.32 *(2.2)*×10^5^	6.90 *(7)*×10^3^	1.74 *(0.64)*×10^6^
**Blood**	4.4 *(4)*	4.01 *(4.0)*	0.301 *(0.3)*	41.8 *(36.4)*

Values in parentheses and italics are corresponding human data for biopsy and blood compartments from Table 3 of Schwartz et al (2011).

**Table 3:** Summary pharmacokinetic parameters in the epithelium and stroma with varying epithelial thickness (h_e_).

	*t_max_* (hr)	*C_max_* (ng/mL)	*C_24 h_* (ng/mL)	*AUC* (ng/mL*hr)
**Epithelium h_e_ = 100 mm**	0.24	3.55E+06	8.55E+02	6.03E+06
**Epithelium h_e_ = 200 mm**	0.72	2.81E+06	6.48E+03	1.07E+07
**Epithelium h_e_ = 400 mm**	1.77	2.09E+06	8.52E+04	1.79E+07
**Stroma h_e_ = 100 mm**	1.53	1.06E+05	2.58E+03	7.04E+05
**Stroma h_e_ = 200 mm**	3.37	1.10E+05	6.93E+03	1.10E+06
**Stroma h_e_ = 400 mm**	7.30	9.52E+04	2.50E+04	1.41E+06

### Results for Daily Dosing and Comparison with Human PK Data

We adapted our model to daily dosing of 4 mL of the 1% Tenofovir gel, and compared results with human PK data from the MTN-001 study [29]. In this analysis, we maintained the assumption of uniform, constant gel coating and reset gel concentration to 1% at 24 hr intervals. The goal of this analysis was to embody the effects of replenishment of gel concentration every 24 hours but not to perform detailed characterization of the extent and effects of gel leakage (which we will perform in a follow up study). Here, we sought to make an initial comparison of model predictions with the second data base of human PK measurements, obtained with a different dosing protocol from the earlier CONRAD study. Results show that concentrations exhibit periodic variations per dosage cycle, beginning after the first cycle, and there are very small quantitative differences in these concentrations across successive dosing cycles. The MTN-001 study has published (Table 3 in reference [29]) a value of 113 ng/mg (i.e. 1.13×10^5^ ng/mL) for median end-of-study Tenofovir concentration across biopsies taken either pre-dose or at 2, 4 or 6 hours following the most recent dose. There is considerable variability in the data that gave rise to this number (cf. Figure 3 in [29]). However it is useful for an initial comparison with our computations of predicted biopsy concentrations for daily dosing. We computed the average concentration in simulated biopsies across 0, 2, 4 and 6 hours to be 1.28×10^5^ ng/mL. This is 13% higher than the median value in the MTN-001 study.

### Effects of Variations in Epithelial Thickness

The thickness of the human vaginal epithelial layer varies in response to cyclic variations in levels of women’s reproductive hormones [23]. In particular, it varies directly with estrogen levels, and is thickest at midcycle. It is also believed the volume of ambient vaginal fluid varies cyclically and is highest at midcycle [28]. Since microbicide gels are intended for application throughout the menstrual cycle, it is biologically important to understand possible consequences of cyclic variability in epithelial thickness and vaginal fluid volume on microbicide drug delivery and PK. In principle, a thicker epithelium would delay the arrival of drug molecules to the stroma, and increased volume of ambient vaginal fluid would increase the extent of gel dilution and potential loss of drug. We used our model to simulate these joint effects of varying epithelium thickness and rate of gel dilution. In these computations, the rate constant *k_D_* for gel dilution was halved for the halved epithelial thickness (100 µm) and doubled for the doubled thickness (400 µm). These variations in *k_D_* derived from a halving and doubling of vaginal fluid volume, respectively, and assuming full dilution in 24 hours. Summary PK measures are given Table 3. We see that as epithelial thickness increases and vaginal fluid dilution decreases, *t_max_* increases, *C_max_* decreases but *C_24 h_* and *AUC* at 24 hours increase That is, there is a delay in drug transport to the stroma, and in concentrations therein and drug delivered at 24 hours becomes higher as epithelial thickness increases.

### Interpreting Concentrations Measured in Biopsy Specimens

As seen in Figure 2, drug concentration varies through the depths of the epithelial and stromal layers in a time-dependent way. Gradients are steeper in the stroma, especially at early times after the onset of drug delivery by the gel. These gradients also depend upon epithelial thickness, which is an order of magnitude smaller than stromal thickness. Drug concentration measured in a biopsy is a spatial average across the epithelial layer and the depth of the stroma that has been excised by the biopsy punch. The thickness of the biopsy varies in practice. Its nominal value in microbicide trials has been 3 mm [44] but in practice it is uncontrolled and can be as low as 2 mm. These variations in both biological epithelial thickness and experimental biopsy thickness influence the interpretation of drug concentrations measured in biopsies. Those concentrations are intended to relate to the biological activity of a drug in its target site. Tenofovir is believed to exhibit its anti-HIV activity primarily (albeit not exclusively) in the stromal layer (where the great majority of host cells are located). Our prior computations (see above) were for a standard biopsy thickness of 3 mm, with an epithelial thickness of 200 µm and a stromal thickness of 2.8 mm in a biopsy that is 3 mm thickness, and show that concentrations in biopsies overestimate concentrations in the stroma. Specifically, we have used the model here to evaluate the relative concentration of Tenofovir in the stroma (where it acts) vs. the concentration of Tenofovir in a simulated biopsy specimen. We again allowed for variations in epithelial thickness and dilution by vaginal fluid, as above, and also varied the thickness of the biopsy specimen from 2 mm to 3 mm. Ratios of summary PK parameters are given in Figure 4. We see here that for thicker epithelial layers, the concentration of drug in the stroma becomes a lower fraction of the drug concentration measured in a biopsy, regardless of biopsy thickness, and is generally less than unity. That is, Tenofovir concentrations in biopsies are predicted to overestimate concentrations in stroma. The differences between biopsy and stromal concentrations are smallest for very thin epithelial layers and biopsies taken at times approaching 24 hours. The overestimation of concentration in stroma increases for thicker biopsies and thicker epithelial layers. For the thickest epithelial layer (400 µm), the biopsy/stromal concentration ratio is about 30% for 3 mm biopsies taken at 4 hours and about 80% for 3 mm biopsies taken at 24 hours; the ratios are about 50% and 95% for 2 mm biopsies at those times.

## Discussion

We have created an initial mechanistic computational model of drug transport from a vaginal gel coating layer into the epithelial and stromal layers of the human vaginal mucosa, with subsequent uptake into the blood stream and lymphatics and clearance there from. This is a continuum model for the time-dependent spatial drug concentration distributions within the gel, epithelial and stromal compartments; concentration in the blood compartment is a time-dependent volume average. The properties of the vaginal compartments are spatially homogenous (drug concentrations therein are not), and the model does not distinguish between paracellular and intracellular drug concentrations. As such, its predictions can be interpreted in relation to measurements in PK studies (e.g. in biopsies), and they were referenced to human PK data in two studies which applied the same 1% Tenofovir gel at 4 mL. The agreement between model predictions and experimental data is encouraging, and suggests that this conceptualization of the mechanisms of TFV delivery by a gel can be useful in understanding the determinants of such delivery. Because of the linear correlation between TFV and TFV-DP concentrations contrasts in Tenofovir concentration predicted here are relevant to our understanding of its anti-HIV functioning in the vaginal mucosa. Moreover, this modeling approach can in principle be extended to characterize TFV phosphorylation to TFV-DP: such a more advanced model would introduce additional terms in the mass conservation equations for TFV, and add coupled equations for conservation of mass of TFV-DP.

Parameters in our model were obtained from multiple sources, including vaginal tissue permeability data; and 3 rate constants were found via fitting of predictions of the model to summary PK measures from a clinical trial [31]. The fitting procedure utilized educated guesses of pharmacologically relevant initial values of the three rate constants. Based on our computational experience with the sensitivity of results to perturbations in these rate constants (data not shown) we therefore believe that the mathematical optimization process did capture a pharmacologically relevant minimum value in the cost function. We note that the model does not include a full triphasic characterization of the blood clearance process; there is a single clearance rate constant deriving from fits of the model to the PK data that were taken over 24 hours after gel insertion. Predictions from these fits to PK measures are reasonable (Table 2). Model predictions were then compared with results from a second clinical trial that used the same gel but a different dosing regimen. Again, reasonable agreement (we predicted a 20% higher value) was obtained. Such agreement does not, of course, constitute full validation of this model – which is proposed as an initial formalism that can and will be improved. Nonetheless, the extent of agreement of model predictions with PK data, together with its mechanistic foundation, do suggest that it can be useful in helping to understand how vaginal gel delivery of Tenofovir is governed by various properties of the drug itself (e.g. diffusion and partition coefficients across all compartments), drug loading into the gel, gel volume and coating of the vaginal mucosa, properties of the epithelial and stromal layers of the mucosa – as they interact with the migrating drug (e.g. thicknesses, clearance kinetics, etc.), and drug clearance from the blood stream. This is a multivariate, non-linear process, and mechanistic modeling can help define the salient parameters that govern it and how their values and interactions affect drug delivery outcomes.

One application of future modeling is to investigate relationships between PK data in animal studies vs. those in the human using allometric scaling. For example, the rabbit and rhesus macaque (used in the other PK studies of the Tenofovir gel [33], [34]) not only have different blood and body masses from women, but also different vaginal sizes, thicknesses and structures of epithelia, and amounts of vaginal fluid. The model here embodies effects of these physiological factors, and thus can be helpful in the scaling for dosing across different species.

Clearly, a logical follow up to this model is to enhance it to include effects on drug transport of time-dependent gel spreading. This is a two dimensional problem that must account for lateral movement of drug – out from the spreading gel into vaginal fluid and within the mucosal layers – as well as movement perpendicular to the mucosal surface. Such analysis will reveal the interaction of gel volume with gel, drug and vaginal properties in governing drug delivery. Gel spreading is relatively fast compared to the 24 hour time scale of the modeling here [45]–[47]. Thus, we expect that the transient effects of gel spreading would tend to be complete within about an hour after gel insertion. This does limit use of the model here in predicting drug concentrations at short times, viz. up to 60 minutes, depending upon gel properties and volume. Nonetheless, for perspective on drug delivery over 24 hours, the perfect coating model here is instructive. For fixed gel thickness, all predicted concentrations are proportional to initial drug concentration in the gel, and t_max_ values do not depend upon it. The 400 µm thickness in the computations corresponds to a gel volume of 4 mL (see Introduction). An instructive contrast is to halve the gel thickness (corresponding to halving its applied volume) but double the initial drug concentration – so that initial mass of drug loaded into the gel is conserved. It follows that in the stroma, *C_max_* increases by 16%, *t_max_* decreases by 26%, *C_24_* decreases by 15% and *AUC* increases by 1%. These results make sense physically: there is more rapid delivery of drug into the stroma (reduced *t_max_* and increased *C_max_*) because of the higher initial concentration in the gel and reduced distance for drug transport out of the gel; however there is also a faster drop off over time in the stroma (reduced *C_24_*) because the gel exhausts its supply of drug sooner; the tradeoff between these effects leads to a slightly increased *AUC* at 24 hours, but faster loss of drug thereafter.

Details of the spatial concentration profile throughout the thickness of the stroma are informative: they delineate conditions for which prophylactic concentrations may exist throughout the entire thickness, vs. whether they occur only in the uppermost portion beneath the epithelial layer. The concentration distribution in the stroma (Figure 2), exhibits steep gradients at early times up to at least 8 hours, so that virtually all drug is in the upper few hundred µm. The distribution becomes relatively flat by 24 hours, at which time values range from about 10^3^–10^4^ ng/mL across the entire stromal thickness, with an average concentration of 6.85×10^3^ng/mL. By 48 hours, the concentration is even flatter across the stroma, and its average value is 2.70×10^2^ ng/mL.

The above distinctions in concentration distributions are influenced by factors that vary during the menstrual cycle, e.g. epithelial thickness and the presence of vaginal fluid. Our model predicted that increasing epithelial thickness (associated with increases in estrogen during the menstrual cycle, i.e. at mid cycle) slowed the transport of Tenofovir to the stroma (and, thus, host cells therein). Doubling epithelial thickness from 200 µm to 400 µm more than doubled the t_max_ value for the stroma from 3.4 to 7.3 hours. Stromal TFV concentration (averaged through its entire depth) at the end of a 24 hour single dose cycle quadrupled from 6.72×10^3^ ng/mL to 25.0×10^3^ ng/mL. The clinical significance of these distinctions is not clear, but they do bear upon how the kinetics of establishing prophylactic drug concentrations in the stroma depend upon epithelial thickness and thus cycle phase. That is, at midcycle it would take longer to establish such concentrations but they would also be maintained longer after gel application.

Our model is also informative about interpretation of concentration values measured in biopsy specimens. Variations in thickness of a biopsy affect the relationship between its drug concentration and that in the stromal compartment (the primary target for TFV). In general, concentrations in biopsies are predicted here to be greater than those in the stroma – by an extent that depends upon interacting effects of biopsy thickness, epithelial thickness and time at which the biopsy is taken (Figure 4). The magnitude of this overestimation is largest for relatively early biopsy times after gel application, where it can be as high as a factor of ∼5 for thicker biopsies and thicker epithelium. The overestimation is smallest for longer times and thinner epithelial layers, regardless of biopsy thickness. Mathematically, it is possible to use our model to provide an algorithm that accounts for time of biopsy by adjusting its drug concentration to an ‘effective’ value that standardizes biopsy timing; this would be based on the time-dependence of predicted concentrations in the mucosal compartments. Such a computational correction could also average over the expected ranges of biopsy and epithelial thicknesses, to achieve further standardization. In any event, the basic results here suggest that there is an inherent uncertainty in the extrapolation of Tenofovir concentration measurements in biopsies to values in the stroma.

In summary, we have introduced a computational model that can be useful in understanding the multiple determinants of microbicide gel drug delivery to the vaginal mucosa. The principles of drug mass transport that are implemented here can be applied to other dosage forms, as well. Of course, some details in such modeling will differ, e.g. in accounting for drug transport within and out from the vehicle, and for the size and shape of the contact surface of the vehicle with the mucosa. Although the general formalism is qualitative, we were able to implement the model quantitatively for the drug Tenofovir, because we could reference its predictions to data from two human PK studies for the same gel. Agreement was, in our view, reasonable. We illustrated use of the model, in evaluating the effects of biological variations in epithelial thickness and vaginal fluid on Tenofovir concentrations in the stroma, and in relating concentrations measured in biopsies to concentrations in the stroma. The model was also used to delineate how drug concentration in the target stromal compartment declined with increasing time after a single gel application, with relevance to consequences of different specified dosing regimens in clinical trials, and of missed gel applications in those regimens. Overall, the results here inform not just our fundamental understanding of intravaginal Tenofovir delivery, but also our ability to design gels and dosage regimens that deliver specified drug concentrations to target locations within the mucosa, over defined intervals after both gel application and exposure to semen-borne HIV.
